# Let's All Speak Together! Exploring the Masking Effects of Various Languages on Spoken Word Identification in Multi-Linguistic Babble

**DOI:** 10.1371/journal.pone.0065668

**Published:** 2013-06-12

**Authors:** Aurore Gautreau, Michel Hoen, Fanny Meunier

**Affiliations:** 1 INSERM, U1028, Lyon Neuroscience Research Center, Brain Dynamics and Cognition Team, Lyon, F-69000, France; 2 CNRS, UMR5292, Lyon Neuroscience Research Center, Brain Dynamics and Cognition Team, Lyon, F-69000, France; 3 University of Lyon, F-69000, France; 4 L2C2, CNRS UMR5304 - Institut des Sciences Cognitives, Lyon, F-69000, France; University of Barcelona, Spain

## Abstract

This study aimed to characterize the linguistic interference that occurs during speech-in-speech comprehension by combining offline and online measures, which included an intelligibility task (at a −5 dB Signal-to-Noise Ratio) and 2 lexical decision tasks (at a −5 dB and 0 dB SNR) that were performed with French spoken target words. In these 3 experiments we always compared the masking effects of speech backgrounds (i.e., 4-talker babble) that were produced in the same language as the target language (i.e., French) or in unknown foreign languages (i.e., Irish and Italian) to the masking effects of corresponding non-speech backgrounds (i.e., speech-derived fluctuating noise). The fluctuating noise contained similar spectro-temporal information as babble but lacked linguistic information. At −5 dB SNR, both tasks revealed significantly divergent results between the unknown languages (i.e., Irish and Italian) with Italian and French hindering French target word identification to a similar extent, whereas Irish led to significantly better performances on these tasks. By comparing the performances obtained with speech and fluctuating noise backgrounds, we were able to evaluate the effect of each language. The intelligibility task showed a significant difference between babble and fluctuating noise for French, Irish and Italian, suggesting acoustic and linguistic effects for each language. However, the lexical decision task, which reduces the effect of post-lexical interference, appeared to be more accurate, as it only revealed a linguistic effect for French. Thus, although French and Italian had equivalent masking effects on French word identification, the nature of their interference was different. This finding suggests that the differences observed between the masking effects of Italian and Irish can be explained at an acoustic level but not at a linguistic level.

## Introduction

In daily life, speech is often produced and perceived with background noise, which interferes with the comprehension of target signals. Similarly, when the background noise is speech, it masks target speech more efficiently than background sounds that are devoid of linguistic content [1], [2]. This efficient masking has been explained in experimental psychoacoustics as the result of the cumulative effect of 2 types of masking phenomena that occur in a cocktail party situation [3]. First, an energetic masking effect is caused by acoustic backgrounds and is due to an overlap at the cochlear level in both the time and frequency between the target and concurrent signals. Second, an additional informational masking effect, which occurs at more central auditory processing stages, is produced by backgrounds that share some information with the target signal [1], [4]. With regard to speech-in-speech comprehension, informational masking involves a competition between different linguistic information levels (i.e., prosodic, phonetic and lexical information) that are extracted from both signals (i.e., target speech vs. concurrent speech). This is of particular interest from a psycholinguistic perspective given that most models of lexical access postulate that word identification is the result of strong competitive mechanisms between simultaneously activated units (see, for example, NAM [5], the revised Cohort model [6], TRACE [7] or Shortlist [8]), although these models have different proposals regarding the exact nature of the competitors. In the present paper, we explore the nature of informational masking phenomena given linguistic information during speech-in-speech recognition. More precisely, the goals of our research are to investigate whether informational masking can be decomposed and, if so, whether it varies depending on the type of linguistic information that is carried by the background speech.

To date, it has been established that the intelligibility of a speaker is rated as higher when the speech is heard with a background of babble spoken in a different language than when the babble is spoken in the same language. This effect has been reported in several studies evaluating the intelligibility of target sentences spoken in English with different babble backgrounds that were either also in English or were from a different language that was unknown to the native English-speaking participants, including Dutch [9], Spanish [10] and Mandarin [11]. Authors explained that when the background babble was spoken in an unknown language, the participants did not understand the concurrent speech, leading to reduced linguistic interference and informational masking effects, which led to improved performance on the task. Other work has further explored this effect by assessing the intelligibility of English target sentences that were masked by 5 different types of backgrounds, each varying in the amount of signal intelligibility [12]. In 2 extreme conditions of intelligibility, the same languages from [11] were used, meaning that English was the identical language and Mandarin was the unknown language. In 3 intermediate conditions, the authors used speech produced by native Mandarin speakers who were speaking English and whose productions were evaluated as having high-, moderate- or low-intelligibility. The authors aimed to test the hypothesis that more intelligible speech in the background would result in the target speech being more difficult to comprehend due to a gradual increase in linguistic interference. At a signal-to-noise ratio (SNR) of −5 dB, the lowest performances were obtained with the native English background, which was the background that was fully intelligible. Regarding the native Mandarin-accented English backgrounds (with high-, moderate- or low-intelligibility), performances significantly increased as background intelligibility decreased. These data suggest that observed differences in the intelligibility of target speech could be explained by the level of intelligibility of the background. In fact, the informational masking effect became more important as the background became more intelligible. This result has also been reported in studies comparing the differential sensitivity of native and non-native listeners of English to energetic and informational compounds of masking during speech-in-speech comprehension [13]. Recent work has examined the intelligibility of English target sentences that were masked by a 2-talker background that was either spoken in the same language (i.e., English) or in a phonetically similar language (i.e., Dutch) [14]. Despite a strong phonetic proximity with the target language, Dutch led to significantly higher performances than English in this study, which is the same pattern of results that was observed when Mandarin was used as the background [11]. In sum, previous studies have shown that language identity (i.e. same as vs. different than the target) and the intelligibility of the background are 2 factors that influence the informational masking effect.

Our current research aims to explore the composition of informational masking by comparing the interference effects of 2 different unknown languages in the same experiment. Until now, studies examining informational masking effects have focused on the level of intelligibility of the background speech. In our work, unintelligible backgrounds are used from 2 languages that are unknown to the participants and that have varying linguistic distances from the target language. Thus, we can examine whether informational masking is composed of different types of linguistic competition, such as phonetic/phonological competition and/or lexical competition. For this purpose, French target words were masked with backgrounds from an identical language as the target speech (i.e., French) or from different languages, which were Irish and Italian. Several linguistic criteria can be used to evaluate the linguistic similarities between languages (see [15]). For our study, we chose the following 2 clear cases in which linguists and non-linguists are likely to agree: Italian is the closer language to French and Irish is the more distant one from French. French is a Romance language, which originated from a distortion of the spoken Latin language from the Roman Empire, as is Italian. Both French and Italian are syllable-timed languages, meaning that every syllable is perceived as lasting approximately the same amount of time, though their absolute durations depend on prosody. In contrast, Irish, also known as Irish Gaelic, is a Goidelic language that originated in Ireland. It is a stress-timed language, in which syllables may last different amounts of time, but a fairly constant duration (on average) is perceived between consecutively stressed syllables. A particularity of Irish words is that they often start with clusters of 2 or 3 consonants (which rarely occurs in French). From a phonological point of view, Italian is much closer to French than Irish is. French is composed of 35 phonemes (14 vowels and 21 consonants). Italian shares 60% of its phonemes with French, whereas only 18% of Irish phonemes are comparable to French phonemes. Overall, Irish sounds very different from French and Italian. We expected that French as the background would provoke a larger masking effect than backgrounds that were spoken in other languages, given that the linguistic content was more similar between the 2 concurrent speech signals. More critically, we evaluated whether the 2 languages that differed from the target speech, which were Irish and Italian, had equivalent informational masking effects. Finally, we examined whether the distance criteria between the languages influenced their masking effects.

We were also interested in dissociating informational masking from energetic masking for all of the backgrounds that were used. To do so, we generated fluctuating noise that had the same energy (i.e., a long-term power spectrum and an envelope below 60 Hz) as our speech backgrounds but did not contain linguistic information. Speech-derived fluctuating noise should lead to better target speech intelligibility than backgrounds containing speech, which produce both energetic and informational masking effects [16], [17]. Comparing the performances obtained with fluctuating noise and speech backgrounds allows us to quantify the informational masking effects of each language (i.e., Irish, Italian and French). Participants were presented with French target words in backgrounds consisting of 4-talker babbles in each language (i.e., Irish, Italian or French) or in fluctuating noise derived from the original 4-talker babbles. During Experiment 1, which was tested at a SNR of −5 dB, participants were asked to write down the target words that they heard. In Experiments 2 and 3, participants performed a lexical decision task. In Experiment 2, the SNR was fixed at −5 dB, whereas the SNR was fixed at 0 dB in Experiment 3.

## Experiment 1: Intelligibility Task at a −5 dB SNR

### Materials and Method

#### Participants

Thirty volunteers participated in Experiment 1. All were native French-speaking students, ages 19 to 30 years, with no knowledge of the 2 foreign languages used in this study (i.e., Irish and Italian). None of the participants indicated having a known hearing loss or language disorder. The present study and the 2 following ones were conducted in accordance with the Declaration of Helsinki. All participants provided written informed consent and were paid for their participation. The protocol used in this experiment was approved by the local ethics committee (CPP Sud-Est IV, Lyon; ID RCB: 2008-A00708-47).

#### Stimuli

The stimuli consisted of 84 different target words mixed with 4 s of background sound (i.e., babble or fluctuating noise).

#### Generating multi-talker babble

For each language used as a background (i.e., Irish, Italian and French), several female and male native speakers were individually recorded in a sound-isolated room. All talkers were asked to read the same passages from the book The Little Prince (In French: ‘Le Petit Prince’), by Antoine de Saint-Exupery, in their native language. This novel was selected because it has been professionally translated and published in a wide variety of languages, including Italian and Irish, and because the vocabulary used in it is accessible to a broad audience, ensuring that it is fully intelligible to most participants in all languages. From all of the recordings, we selected 2 female and 2 male talkers who had the most natural and standard speaking styles (i.e., no exaggerated prosody, no overplay and no marked regional accents) for each language. Selected recordings were modified according to the following protocol: (i) removal of silences and pauses exceeding 500 ms, (ii) removal of sentences containing pronunciation errors or proper nouns and (iii) intensity calibration at 70 dB-A. Given that auditory stream segregation, particularly for speech-in-speech situations, is primarily based on pitch (F0) information [4], we wanted to equalize this parameter across languages. To avoid important differences in vocal characteristics between the different talkers who were selected for each language, the fundamental frequencies (F0) for each of the 4 talkers in each language were normalized to the closest target F0 values among the 4 values as follows: 205 Hz and 225 Hz (2 female voices) and 105 Hz and 125 Hz (2 male voices). These F0 modifications were performed using the built-in pitch manipulation tool in PRAAT [18]. The babbles consisted of 4-talkers given that past research from our group [2] and from others [4] has demonstrated that this particular listening situation, in which there is 1 target voice and 4 masker voices, allows for the differentiation of the informational and energetic compounds of masking in speech-in-speech. Finally, to avoid “frozen noise” phenomena [19], [20], which is caused by multiple presentations of the same noise sample, 42 sequences of 4 s each were randomly extracted from each recording, and the 4-talker babbles were generated by mixing 1 randomly chosen sequence of 4 s from each of the 4 talkers in 1 language. Fourteen different 4-s-long sequences of the 4-talkers babble were ultimately created for each language, leading to a total of 42 4-talker babble samples.

#### Generating fluctuating noise

To obtain fluctuating noise with comparable energetic masking characteristics as those for the babbles, we derived 42 fluctuating noise samples directly from the 42 samples of the 4-talker babbles that were previously mentioned, according to the following protocol: Using MATLAB© (R2010a, The MathWorks, Inc., Natick, Massachusetts, USA), we first computed the energy root mean square (rms) of the original sample and extracted its temporal envelope by applying a 60 Hz low-pass filter. Then, a fast Fourier transform (FFT) was used to extract the power spectrum and phase distribution of the original signals. The original phase distribution was randomized, and the original modulation was reintroduced by multiplying the random-phased signal by its original envelope. Finally, an inverse FFT was used to generate new signals that were normalized to the rms power of the original sample.

#### Target words

Target words, all of which were in French, were recorded by a female native French speaker and differed from those used in the babbles. Eighty-four French disyllabic words were selected with a middle range of frequency of occurrence (ranging from 0.29 to 175.65 per million; mean  = 17.16, SD  = 30.43), according to the Lexique2 database [21]. This range was selected to avoid extremely high- and low-frequency items that the participants may typically overuse or use too seldom.

#### Stimuli and word lists

Target words were inserted 2.5 s from the start of a 4-s-long background sample at a SNR of −5 dB. Thus, all participants had the same duration of exposure to the background sound before a target word was presented. Among the 84 stimuli, 6 were used as practice items, representing the 6 conditions (2 background types (babbles vs. fluctuating noise) * 3 languages (Irish vs. Italian vs. French)). To ensure that each of the 78 remaining words was presented in each of the 6 conditions, 6 different experimental lists were generated. For example, in list 1, the target word “ballon” was presented in the condition “babble in Irish”. In list 2, “ballon” was presented in the condition “babble in Italian”. In list 3, it was presented in the condition “babble in French”. In lists 4, 5 and 6, it was presented in the conditions “Irish fluctuating noise”, “Italian fluctuating noise” and “French fluctuating noise”, respectively. Therefore, across lists, each of the 78 words was presented in all of the conditions. Each participant heard only 1 list, such that each target word was only presented once to a participant to avoid repetition effects. Within each list, the order of the stimuli was randomized across participants to avoid presentation order effects.

#### Procedure

Participants were tested individually. They sat in a quiet room facing a computer monitor. Stimuli were delivered with DMDx [22] diotically via headphones (Sennheiser HD 448) at a comfortable sound level. The output (i.e., a target word inserted in a background sound) was fixed at 65 dB SPL, as measured with an artificial ear. The task for participants consisted of transcribing a single word, such that they were asked to write down the target word that they had heard. Participants could listen to each stimulus no more than once, and they moved from trial to trial by pressing the space bar on a keyboard. Before the testing phase, participants were given 6 practice items (each of the 6 words appeared in one of the 6 conditions) to familiarize themselves with the stimulus presentation mode and the target voice. The experiment lasted approximately 30 min.

### Results

Participants’ answers were analyzed for correct word identification rates by calculating the proportion of transcribed words that corresponded to the target words. Spelling errors were not taken into account. Raw intelligibility scores were converted into rationalized arcsine units (RAU) for statistical analyses [23]. A repeated measures analysis of variance (ANOVA) was conducted with RAU scores as the dependent variable and background (babble vs. fluctuating noise) and language (Irish vs. Italian vs. French) as the within-subjects factors. For clarity, we use the percentages of correct responses when describing or graphically representing the data.

**Figure 1:**
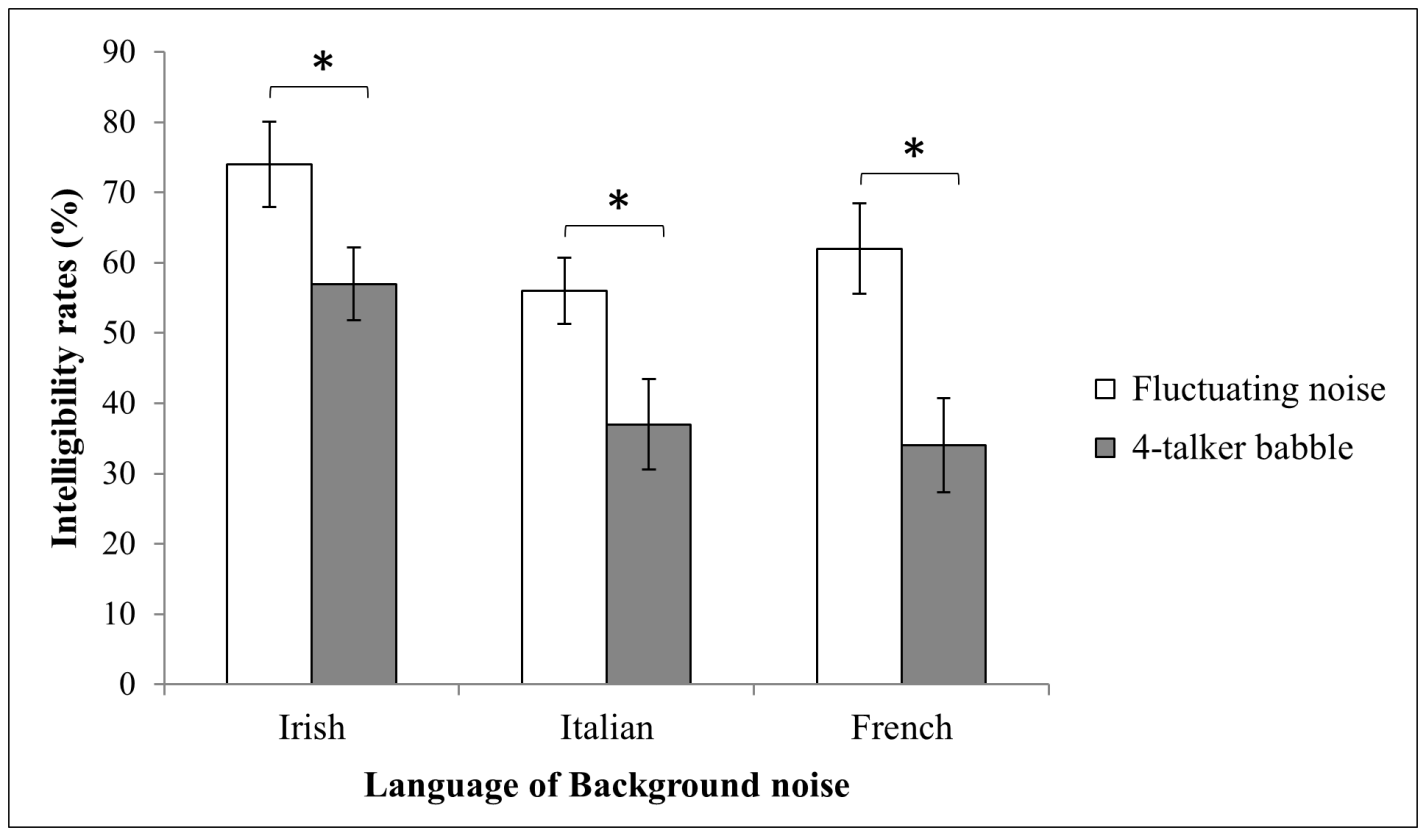
Intelligibility rates (%) at a −5 dB SNR. The intelligibility rates (%) were obtained at a −5 dB SNR for the two types of background (babble vs. fluctuating noise) depending on language (Irish vs. Italian vs. French). Standard errors are reported. The symbol ‘*’ indicates a significant difference between the babble and fluctuating noise conditions in Irish, Italian and French.

The analysis revealed a significant main effect of background (F(1,29) = 149.51, p<.0001) (Figure 1). On average, performances were lower with babble (43%) than with fluctuating noise (64%). A significant main effect of language also emerged (F(2,58) = 41.74, p<.0001). Overall, Irish backgrounds led to better performances (65%) than French (48%) or Italian (46%) backgrounds. The interaction between these 2 factors was significant (F(2,58) = 3.35, p<.05). Post-hoc comparisons with the HSD Tukey test showed that the intelligibility scores obtained with fluctuating noise derived from Irish and Italian (p<.001) and between Irish and French (p<.001) were significantly different. Significant differences also emerged between Irish and Italian (p<.001) and between Irish and French (p<.001) babble backgrounds. For each language, the effect of the background was significant, with higher target word intelligibility ratings in fluctuating noise than in babble.

### Discussion

The intelligibility of the French target words was influenced by the language of the babble. In this speech-in-speech situation, some languages interfered more with the French target words than others. This was the case with French and Italian, which both had stronger masking effects than Irish. Italian and French babbles led to equivalent intelligibility. In other words, Italian, which is different from the target language, had an equivalent masking effect to the same language as the target language (i.e., French). This result was not observed for the Irish language background, which had significantly better intelligibility with regard to the target words. As a consequence, these 2 different languages (i.e., Irish and Italian) did not hinder the intelligibility of the French target words equally, as the closer language (i.e., Italian) had a stronger masking effect than the more distant language (i.e., Irish). For each language, significant differences were observed between performances with 4-talker babbles and those with fluctuating noise. The intelligibility of the target words was always lower with babbles than with fluctuating noise. The linguistic information that was associated with the acoustic information in the babbles masked target speech more effectively than the acoustic information alone in the fluctuating noise. This result highlights the linguistic effect of each language.

Experiment 1′s intelligibility task allowed for the quantification of the intelligibility of the French target words in each listening condition. To measure the online competition between the target speech and the background speech more directly, we tested the same experimental conditions but with a lexical decision task, the validity of which has already been demonstrated with speech-in-speech situations [24]. Participants had to respond as quickly as possible regarding whether the target item was a word, such that their reaction times could provide information about latencies of lexical access. In this second experiment, the speed of lexical access was measured to test the effects of the nature of the background (babble vs. fluctuating noise) and the masking language (Irish vs. Italian vs. French). Longer reaction times to reach a decision would suggest a greater complexity of lexical access due to more interference between the target and concurrent speech signals.

## Experiment 2: Lexical Decision Task at a −5 dB SNR

### Materials and Method

#### Participants. Speakers

In Experiment 2, the 4-talker babble signals were identical to those used in Experiment 1. The target items (i.e., words and pseudowords) were produced by a different female native speaker of French than the speaker for the French babble.

#### Listeners

Thirty volunteers, who had not participated in Experiment 1, participated in Experiment 2. They were all native French-speaking students, ages 18 to 27 years, with no knowledge of the foreign languages used in this study. None of the participants indicated having a known hearing loss or language disorder. They signed informed consent forms and were compensated for their participation.

#### Stimuli. Masker sounds

Hundred and sixty-two maskers (i.e., 4-talker babbles in Irish, Italian and French, and speech-derived modulated noises) were created, according to the same procedure as in Experiment 1.

#### Target items

Eighty-one French disyllabic words and 81 pseudowords constituted the target items. The 81 words were selected from the 84 words used in Experiment 1. All of the pseudowords were consistent with French phonotactic rules, for example, *trouchet*.

#### Stimuli and word lists

The 162 stimuli consisted of 81 target words and 81 target pseudowords, which were mixed with 4 s of background sound (i.e., babble or fluctuating noise) at a SNR of −5 dB. Stimuli were generated in the same manner as in Experiment 1. Target items were inserted at 2.5 s from the start of a background sound. Of the 162 stimuli, 6 were used as practice items (3 words and 3 pseudowords). To ensure that each of the 156 remaining words or pseudowords was presented in each of the 6 conditions (2 background types (babble vs. fluctuating noise) * 3 languages (Irish vs. Italian vs. French)), 6 different experimental lists were generated. As in Experiment 1, across the lists, each of the words or pseudowords was presented in all of the conditions. Each participant heard only one list, such that each target word or pseudoword was presented only once to each participant to avoid repetition effects. Within each list, the order of the stimuli was randomized across participants to avoid presentation order effects.

#### Procedure

The procedure was the same as in Experiment 1, except that E-prime software was used to present the stimuli (Psychology Software Tools, Inc., Pittsburgh, PA, USA). Participants were instructed to perform a lexical decision task with the target items that were inserted against the background sounds. Participants’ task was to decide as quickly and accurately as possible whether the target item was a word by pressing 1 of 2 pre-selected keys on a computer keyboard. Prior to the testing phase, participants were given 12 practice items (each of the 3 words and 3 pseudowords appeared with the 2 types of background sounds in one of the 3 languages) to familiarize themselves with the stimulus presentation mode and the target voice. The experiment lasted approximately 30 min.

### Results

Two analyses of variance (ANOVA) were conducted on the performances of the participants at a −5 dB SNR. For the first analysis, mean reaction times (RTs: time-interval in milliseconds between the onset of the target speech and the participants’ button press) for the correct responses to the target words in each experimental condition were measured. Trials in which participants made mistakes (34.1%), provided no response during the allotted time of 4,500 ms (4.3%), or had RTs lower than 300 ms (0.2%) were not included in this analysis. Thus, the first analysis included RT as the dependent variable and background (babble vs. fluctuating noise) and language (Irish vs. Italian vs. French) as the within-subjects factors. A second analysis of variance included error rates as the dependent variable, with the within-subjects factors the same as in the first analysis.

**Figure 2:**
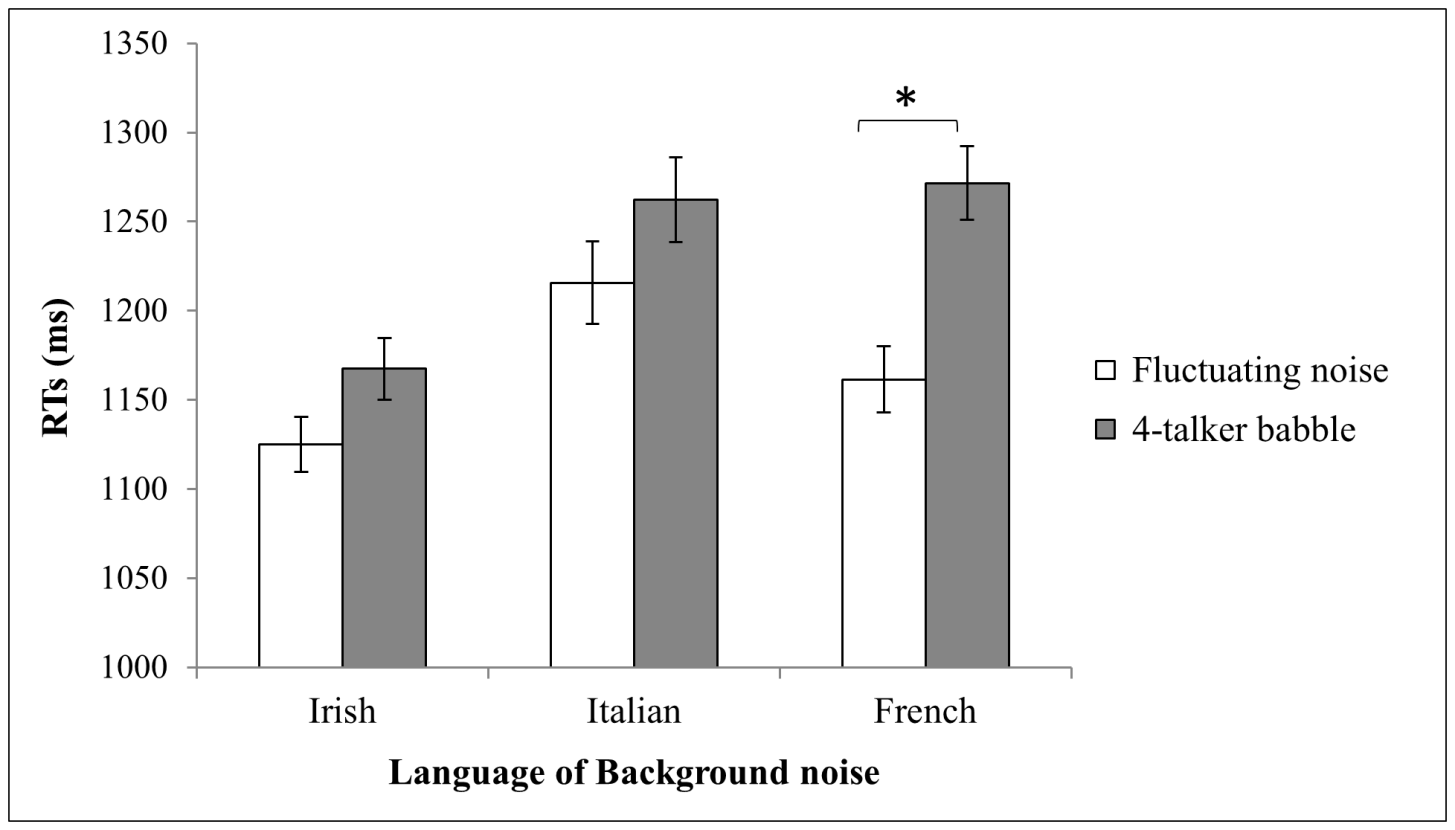
Mean reaction times (ms) for target word identification at a −5 dB SNR. Mean reaction times (ms) for target word identification were obtained at a −5 dB SNR for the two types of background (babble vs. fluctuating noise) depending on language (Irish vs. Italian vs. French). Standard errors are reported. The symbol ‘*’ indicates a significant difference between the babble and fluctuating noise conditions in French.

The first analysis conducted on RTs (Figure 2) revealed a significant main effect of background (F(1,29) = 8.92, p = .005). Mean RTs were longer when the background was composed of babble (1,234 ms) compared to when it was fluctuating noise (1,167 ms). The results also revealed a significant main effect of language (F(2,58) = 5.83, p<.005). Descriptively, participants were faster when the background was Irish (1,146 ms) and slower when it was Italian (1,217 ms). Participants were slowest when the background was French (1,239 ms). The interaction between these two factors was not significant (F(2,58) = 1.25, p = .29). Post-hoc comparisons with the HSD Tukey test showed that there were no significant differences between the languages when the background was fluctuating noise. However, when the background included babble, a tendential difference emerged between Irish and Italian (p = .07), and there was a significant difference between Irish and French (p<.05). Finally, the effect of background was present only for French (p = .02), with RTs significantly faster when the background was fluctuating noise compared with when it was babble.

The second analysis of variance with error rates as the dependent variable indicated a significant main effect of background (F(1,29) = 5.49, p<.05). Error rates were significantly higher when the background included babble (mean = 37%; S.D. = 17.4%) than when it was fluctuating noise (mean = 31%; S.D. = 14.8%). The results also revealed a significant main effect of language (F(2,58) = 9.29, p<.001). On average, the error rates were significantly lower when the backgrounds were Irish (mean = 30%; S.D. = 15%) and French (mean = 31.7%; S.D. = 14.9%) than when it was Italian (mean = 40.5%; S.D. = 17.4%). The interaction between these 2 factors was not significant (F(2,58) = .52, p = .6). Post-hoc comparisons with the HSD Tukey test revealed that when the background contained fluctuating noise, there was a significant difference between French and Italian (p = .04) and a tendential difference between Irish and Italian (p = .06). When the background was babble, there was only a significant difference between Irish and Italian (p<.005).

### Discussion

As in Experiment 1, the language of the babble influenced the participants’ performance. In fact, lexical access of the French target words was significantly longer when the backgrounds were French and Italian than when it was Irish. These results suggest that the 2 languages that were different from the target language (i.e., Italian and Irish) did not have equivalent masking effects. Italian had identical informational masking effects as French, whereas participants were significantly faster when the background was Irish. Again, the closer language (i.e., Italian) had a stronger masking effect than the more distant language (i.e., Irish). When examining the error rates, we found that the Italian language was associated with more lexical decision errors than the 2 other languages. This effect could be attributed to an informational ambiguity effect that is caused by the proximity of the Italian language to the French target words. When Italian is used as the background language, it directly interferes with French targets and causes words or pseudowords to be difficult to differentiate. This leads the error rate to be maximal, and merely above chance, for a background consisting of Italian babble (44%). However, the difference between French and Italian is significant only with a derived modulated noise background, suggesting that a large part of this effect must be explained by acoustic characteristics that are specific to Italian or to the Italian speakers we selected. Amplitude modulations, speech rate, the duration of the available gaps in the background and other prosodic cues could have interfered with the lexical decisions being performed regarding the French items.

In general, the results obtained with fluctuating noise in the intelligibility task (Experiment 1) were not completely confirmed by those obtained with the lexical decision task (Experiment 2). In the intelligibility task, significant differences between performances in babble and fluctuating noise for each language were observed, suggesting that the linguistic and acoustic information contained in the French, Italian and Irish babble was hindering target speech intelligibility. In the lexical decision task, the masking effect of some of the languages seemed to only have an acoustic origin. For Italian, performances in the Italian-derived fluctuating noise and babble backgrounds were not significantly different. This lack of a difference suggests that the linguistic information contained in Italian did not contribute to the informational masking of the target speech as much as it did in the intelligibility task, in which the acoustic information played a more an important role. In contrast, for French, the informational masking of the target words was significantly more important when the background was babble than when it was fluctuating noise, which highlights the effect of the linguistic information contained in French. This observation suggests that the 2 languages, which led to similar performances, in fact, had informational masking effects that were of different origins. Similar to Italian, Irish had an acoustic masking effect, given that no difference between the 2 types of maskers was observed. The difference in masking effects observed between Italian and Irish was at an acoustic level. Overall, in this experiment, the 2 languages differing from the target language primarily had acoustic masking effects on the target speech, and linguistic interference was only present when the target and concurrent speech were the same. In this experiment, the lexical decision task led to a high error rate (34.1%) compared to the error rates of approximately 10% that are typically observed in lexical decision experiments that are conducted in quiet, suggesting that there are difficulties that need to be addressed with this task. Therefore, to test the interaction between the masking effects and task complexity, we decided to conduct a third experiment in which we used the same experimental conditions as in Experiment 2 but at the more favorable SNR of 0 dB.

## Experiment 3: Lexical Decision Task at a 0 dB SNR

### Materials and Method

#### Listeners

Twenty-eight volunteers, who had not participated in either Experiments 1 or 2, participated in Experiment 3. They were all native French-speaking students, ages 18 to 30 years, with no knowledge of the foreign languages used in this study. None had indicated having a known hearing loss or language disorder. They signed informed consent forms and were compensated for their participation.

#### Stimuli

Experiment 3 used the same experimental material as Experiment 2 but the target items were inserted in the various background noises at a SNR of 0 dB. All other procedures, materials, and methods were identical to Experiment 2.

### Results

Two ANOVAs were conducted on the performances of the participants at a 0 dB SNR. For the first analysis, mean RTs for the correct responses to the target words in each experimental condition were measured. Trials in which participants made mistakes (22.1%), provided no response during the allotted time of 4,500 ms (0.8%), or had RTs that were lower than 300 ms (0.2%) were not considered. The first analysis included RT as the dependent variable and background (babble vs. fluctuating noise) and language (Irish vs. Italian vs. French) as the within-subjects factors. The second ANOVA included error rates as the dependent variable, with the within-subjects factors the same as in the first analysis.

**Figure 3:**
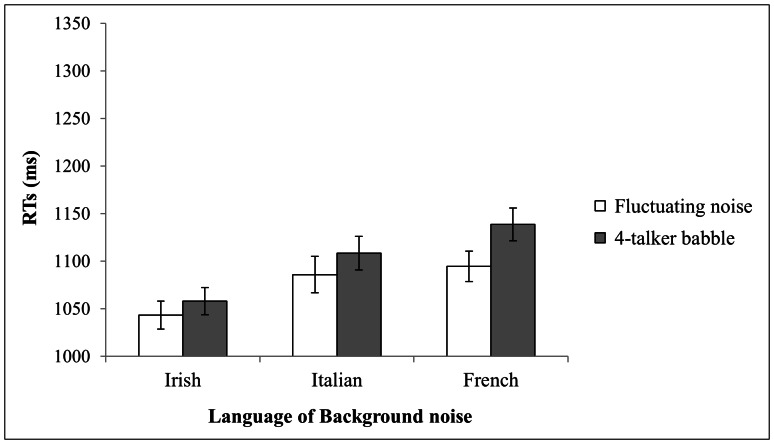
Mean reaction times (ms) for target word identification at a 0 dB SNR. Mean reaction times (ms) for target word identification were measured at a 0 dB SNR for the two types of background (babble vs. fluctuating noise) depending on language (Irish vs. Italian vs. French). Standard errors are reported.

In Experiment 3 (Figure 3), the first analysis conducted on RT revealed that the main effect of background was not significant (F(1,27) = 3.13, n.s.). Statistical analysis indicated a significant main effect of language (F(2,54) = 7.12, p<.001). On average, participants were faster with Irish in the background (1,051 ms) and significantly slower with Italian (1,097 ms) and French (1,117 ms) in the background. The interaction between these 2 factors was not significant (F(2,54) = 0.3, p = .73).

The second analysis conducted on error rates indicated a significant main effect of language (F(2,54) = 5.89, p<.005). On average, error rates were lower with Irish (mean = 19.2%; S.D. = 12.8%) and French (mean = 20.3%; S.D. = 13.1%) in the background and significantly higher with Italian (mean = 26.6%; S.D. = 12.7%) in the background. The interaction between these factors was not significant (F(2,54) = 1.3, p = .28). Post-hoc comparisons with the HSD Tukey test showed that, with fluctuating noise as the background, there was a significant difference between French and Italian (p = .04) and a tendential difference between Irish and Italian (p = .06).

### Discussion

At a 0 dB SNR, no difference emerged between the intelligibility of the babble and fluctuating noise, suggesting that the observed main effect of language could at least partly be explained by differences in the acoustic properties of the languages. This was confirmed when examining error rates, given that we found a main effect of language without a main effect of background. Italian was associated with more errors than the other 2 languages were at −5 dB SNR. This rules out an explanation considering increased informational masking due to the proximity of the Italian language to the French language. At a 0 dB SNR, acoustic characteristics specific to the Italian language or to the Italian speakers we selected caused more interference than the French and Irish backgrounds. All of these results suggest that, at a 0 dB SNR, the segregation between babble and target speech is easier in that there is a reduction in the informational masking of a phonological or lexical origin, which leaves the opportunity for masking that is due to language- or speaker-specific acoustic/prosodic masking.

## General Discussion

In the current study, we were interested in speech-in-speech comprehension and, specifically, in the informational interference that can occur in this type of listening situation. The goal was to directly compare the masking effects produced by languages that differed from the French target words, in this case, Irish and Italian, with the masking effect produced by the identical language, i.e., French. Experiment 1, at −5 dB SNR, examined this question using an intelligibility task, whereas in Experiments 2, at −5 dB, and 3, at 0 dB, the paradigm was a lexical decision task.

### Masking effects of Irish, Italian and French babble

For the speech-in-speech situation, the intelligibility task and the lexical decision task presented at −5 dB SNR revealed similar patterns of results. The babble produced in the different languages (i.e., Irish, Italian or French) hindered French target word intelligibility to varying degrees. First, the listening situation appeared difficult when the background was spoken in the language that was identical to the target speech (i.e., the French-in-French situation). This result is consistent with previous research with English native listeners and English as the target language. For example, a decrease in successful identification of English target sentences was reported in 2-talker babble spoken in English compared to Mandarin [11] (see also [25], with Croatian 2-talker babble). These studies used backgrounds composed of 2-talker babble in which each individual talker can be followed independently. An important decrease in masking can thus be obtained by clearly separating the 2 auditory streams by processing the pitch information from both talkers’ voices, which may reduce the informational masking effects [4]. In our study, we focused on cases in which individual voice characteristics are less predominant, i.e., 4-talker babbles. In this situation, the identical language may have led to a strong masking effect, demonstrating that it is relevant to study linguistic interference in multi-talker and multi-linguistic cocktail party situations. Despite the fact that the energetic masking caused by a 4-talker babble is stronger than the one caused by a 2-talker babble, because the addition of talkers to the babble leads to progressive spectro-temporal saturation due to a shrinking of the temporal window that is available for listening to target words [17], the informational masking effect of the French babble still led to decreased performances. The results of the present study are consistent with those obtained in an intelligibility task with French target words that were masked by concurrent French babble produced by 4-, 6- or 8-talkers [2]. The authors observed that the intelligibility of the target words was most hindered with 4-talkers, demonstrating that this condition causes an increase in informational masking. In this condition, and not with the 6- or 8-talker babbles, some words from the babble were identified as target words. This shows that even if the 4-talkers in the background could not be followed, some information from the background noise, such as word identity, was still available. Having more than 4-talkers leads to an energetic masking effect that overpowers the informational masking effect. Previous research has shown a significant difference in the intelligibility of English target sentences between 2-talker babbles in English and Mandarin, but these linguistic effects did not appear for 6-talker babbles [11]. Our results show that linguistic processes of background words are still achieved with up to 4-talkers.

Second, when the language in the background differed from that of the target words, the background languages had differential effects on intelligibility and lexical decisions. To date, studies that have manipulated the language in the background speech have reported that a background in a different language than the target language always leads to lower masking effects than a background that is spoken in the identical language as the target language. Our experiments, which tested the masking effect of 2 languages that were unknown to the participants, revealed that masking effects varied depending on the foreign language spoken in the background. In our case, Irish and Italian did not lead to equivalent performances, and the expected results were found only for Irish, as its masking effect was significantly lower than that of the French background (i.e., the identical language). The Italian background hindered target word identification as much as the French background did. These results suggest that the language that was closer to French, i.e., Italian, had a stronger masking effect than the language that was more distant from French, i.e., Irish.

A limitation of many multi-language studies is that the talkers differ from one language to another and, therefore, there are differences in the acoustical characteristics, such as in spectral energy, in their recordings. Consequently, the following 2 factors are confounded: the languages of the babble and the acoustical characteristics related to the language and talkers. In a recent intelligibility task with English and Dutch target sentences, acoustical differences observed between the 2-talker babbles in Dutch and English were minimized by producing a LTAS (long-term average spectrum) normalization of the 2-talker babbles [14]. Thus, the differences in the amount of energetic masking were reduced. In our experiments, the influence of acoustics that were specific to a language (e.g., accentuation pattern or phonotactics) or talker (e.g., speaking rhythm or prosody), was isolated from the influence of higher order linguistic effects (i.e., phonological or lexical) through the use of matched fluctuating noise, which provided a within-language control for the energetic/informational factors.

### Nature of the Interference

The results obtained using the different languages as backgrounds informed us about which language had the most detrimental effect on the comprehension of French target words. Analyzing the performances with fluctuating noise allowed us to further explore the nature of the interference observed for each language. Fluctuating noise was generated to have similar spectro-temporal and envelope information as babble but without the phonological and lexical information. By comparing performances with babble and fluctuating noise as the backgrounds, it is possible to evaluate the contribution of the phonological or lexical information of each language to informational masking. However, the intelligibility and lexical decision tasks did not reveal the same pattern of results regarding the nature of this interference.

The intelligibility task (Experiment 1) revealed linguistic interference for Irish, Italian and French, whereas the lexical decision task (Experiment 2) revealed that only French had a linguistic masking effect. In Experiment 2, no significant differences were observed between the conditions with Irish and Italian babble in the background and the ones with the corresponding fluctuating noise in the background. This observation suggests that linguistic information did not hinder target word identification speed and that only the acoustic component of the fluctuating noise could account for the observed interference effect. Specifically, when analyzing the error rates in the lexical decision task, we found that language- or speaker-specific acoustic characteristics of the Italian language caused a particularly large masking effect. This observation suggests that the informational masking associated with the speech-in-speech situation is composed of at least 2 different origins: a first level of interference that is caused by lower level linguistic information, such as rhythmic or prosodic information, and a second level of interference that engages higher level phonological or lexical information. These 2 levels can be separated using fluctuating speech-derived noises. Second, this observation suggests that these different levels will interfere depending on their availability at the cognitive level, such that higher-order interference disappears when the SNR becomes null. Additionally, this depends on the task being use, as higher-order (phonological/lexical) interference is mainly observed in the intelligibility task, whereas lower-order (acoustic/prosodic) interference plays a major role in the lexical decision task (see [26] for data on language rhythm).

This difference in results between the 2 tasks is a reflection of differences in the cognitive processes that they capture. The lexical decision task is an online measure (reaction times), whereas the intelligibility task leaves some time for post-processing (accuracy). Therefore, these 2 tasks are differentially sensitive to the 2 types of informational interference. On one hand, the intelligibility task is described as offline, meaning that comprehension occurs after the perceptual processes involved in lexical access have been executed. In this task, participants wait until the end of the background noise to write down the target word as they perceived it. Thus, a certain amount of time elapses between the end of the presentation of the target word and the end of the trial. During this interval, post-lexical processes, such as influences on decision making that are due to explicit knowledge about language and metalinguistic abilities, may occur and modify the participants’ final responses. On the other hand, the lexical decision task is considered an online task in which participants provide their responses as quickly as possible once they have identified whether the target item was a word. This task can capture the competition that occurs during lexical access, given that the influence of post-lexical processes is limited. The results from the lexical decision task revealed that lexical access was disrupted only when French was in the background, and this result was not evident for Italian or Irish. This finding suggests that the linguistic effect of Irish and Italian that was observed in the intelligibility task results from post-lexical processes that influence decision making and take higher-order linguistic information, such as phonological plausibility or lexical predictions, into account.

The result showing no such interference caused by Irish and Italian in the lexical decision task suggests that this online task is not sensitive to these interferences. On the contrary, it may be that only low-level prosodic information influenced lexical access. Thus, the phonemes that Irish and Italian have in common with French did not compete with the French phonemes during lexical decisions. This result is supported by studies showing that the production of a given phonemic contrast varies at the articulatory level according to the language in which it is produced, leading to differences in the perception of this contrast [27], [28]. In our case, phonemes from the various languages did not compete with each other, even if they were categorized similarly (i.e., according to the International Phonetic Alphabet), which may be because they were perceived as being different. This would support the importance of fine acoustic details during speech perception. For a long time, traditional psycholinguistic models, such as TRACE [7] and Shortlist [8], have considered the processes underlying the mapping of sensory information from the acoustic input to stored entries in the lexicon from a phonemic approach, according to which the signal is converted into phonemes through the loss of fine acoustic details. A growing amount of data suggests that fine acoustic details are taken into account during speech processing (see, for example, [29]). Our results suggest that the fine acoustic details that differentiate the production of a phoneme in different languages can be used to prevent interference during lexical access.

### Conclusions

In this paper, comparing an offline measure (i.e., an intelligibility task) and an online measure (i.e., a lexical decision task) allowed us to further explore the masking effects of the identical language to the target language, which was French, and of different languages (i.e., Irish and Italian). Both tasks revealed significantly divergent results for the different languages, i.e., the speech backgrounds produced in Italian hindered intelligibility of French target words to a similar extent as those in the identical language (i.e., French), whereas the Irish speech backgrounds supported the best performances. Moreover, the lexical decision task at a −5 dB SNR was better for exploring our measures than the intelligibility task at the same SNR, given that the lexical decision task minimized post-lexical processes and allowed us to dissociate acoustic from linguistic masking effects for the various languages being studied. Specifically, acoustic and linguistic masking effects were revealed for the identical language (i.e., French), whereas only acoustic masking effects were observed for the different languages (i.e., Irish and Italian). This finding suggests that the linguistic distances from Irish and Italian to French did not influence participants’ performances and that French and Italian, i.e., identical vs. different language to the target language, had masking effects of equal strength but differing in nature.
